# Leaf dynamics in growth and reproduction of *Xanthium canadense* as influenced by stand density

**DOI:** 10.1093/aob/mcv114

**Published:** 2015-08-05

**Authors:** Takahiro Ogawa, Shimpei Oikawa, Tadaki Hirose

**Affiliations:** Department of International Agricultural Development, Tokyo University of Agriculture, Tokyo 156-8502, Japan

**Keywords:** Leaf dynamics, leaf longevity, leaf productivity, mean residence time, nitrogen resorption, nitrogen use efficiency, stand density, *Xanthium canadense*, cocklebur, Asteraceae

## Abstract

**Background and Aims** Leaf longevity is controlled by the light gradient in the canopy and also by the nitrogen (N) sink strength in the plant. Stand density may influence leaf dynamics through its effects on light gradient and on plant growth and reproduction. This study tests the hypothesis that the control by the light gradient is manifested more in the vegetative period, whereas the opposite is true when the plant becomes reproductive and develops a strong N sink.

**Methods** Stands of *Xanthium canadense* were established at two densities. Emergence, growth and death of every leaf on the main stem and branches, and plant growth and N uptake were determined from germination to full senescence. Mean residence time and dry mass productivity were calculated per leaf number, leaf area, leaf mass and leaf N (collectively termed ‘leaf variables’) in order to analyse leaf dynamics and its effect on plant growth.

**Key Results** Branching and reproductive activities were higher at low than at high density. Overall there was no significant difference in mean residence time of leaf variables between the two stands. However, early leaf cohorts on the main stem had a longer retention time at low density, whereas later cohorts had a longer retention time at high density. Branch leaves emerged earlier and tended to live longer at low than at high density. Leaf efficiencies, defined as carbon export per unit investment of leaf variables, were higher at low density in all leaf variables except for leaf number.

**Conclusions** In the vegetative phase of plant growth, the light gradient strongly controls leaf longevity, whereas later the effects of branching and reproductive activities become stronger and over-rule the effect of light environment. As leaf N supports photosynthesis and also works as an N source for plant development, N use is pivotal in linking leaf dynamics with plant growth and reproduction.

## INTRODUCTION

Because light not only is the energy source for photosynthesis but also has several photomorphogenetic effects (Lambers *et al*., 1998; Jones, 2014), it strongly influences the size and form of the plant. Plants growing in an open habitat typically have a short erect stem and develop many branches, while plants growing in crowded habitats increase height and show less branching (Bazzaz and Harper, 1977; Weiner *et al*., 1990; Nishimura *et al.*, 2010; Watari *et al*., 2014). Such changes have been considered adaptive in the sense that the plant would maximize fitness relative to neighbours by capturing light before others use it (Givnish, 1982, 1995; Sakai, 1991; Schieving and Poorter, 1999; Falster and Westoby, 2003; Anten, 2005; Hikosaka and Anten 2012). To use light and nitrogen (N) for photosynthesis efficiently, plants allocate N among leaves such that leaves receiving the highest light have the highest N per leaf area (Field, 1983). Canopy photosynthesis is maximized by the distribution of leaf N allocated according to the gradient of light interception in the canopy (Hirose and Werger, 1987; Farquhar, 1989; Pons *et al*., 1989; Werger and Hirose, 1991; Schieving *et al*., 1992; Anten *et al*., 1995; Niinemets *et al*., 2014). *Lysimachia vulgaris* maintained a greater leaf mass with a shallower slope of leaf N in an open than in a dense stand (Hirose *et al*., 1988). Leaves of a vine, *Ipomoea tricolor*, changed leaf N per area depending on irradiance irrespective of leaf age (Hikosaka *et al.*, 1994; see also Ackerly 1992). Resorption and reallocation of N from shaded old leaves to sun-lit new leaves may increase whole-plant carbon gain (Franklin and Ågren, 2002; Escudero and Mediavilla, 2003; Hikosaka, 2003; Oikawa *et al*., 2008; Marty *et al*., 2010). Different leaf dynamics are then expected between plants in an open and in a dense stand. Here leaf dynamics implies changes in leaf mass and life expectancy determined by the balance between leaf birth and death (Bazzaz and Harper, 1977; Chabot and Hicks, 1982; Kikuzawa, 1983; Harper, 1989; Ackerly and Bazzaz, 1995; Hikosaka, 2005).

Hirose and Oikawa (2012) demonstrated that mean residence time (MRT, d) defined for leaf number, area, dry mass and N (collectively ‘leaf variables’) was useful for the study of leaf dynamics. The concept of MRT was introduced earlier as one of the two factors comprising plant N use efficiency (NUE, g g^−1^ N; Berendse and Aerts, 1987) and defined as the expected length of time plant N is retained in the plant body before being lost. The other factor was N productivity (NP, g g^−1^ N d^−1^; Ingestad, 1979), i.e. dry mass productivity per unit plant N. Mean residence time of plant N has been calculated from the mean standing plant N divided by the amount of N lost in a year, assuming implicitly that the N flux through the plant was at a steady state (Garnier and Aronson, 1998; Aerts and Chapin, 2000; Eckstein and Karlsson, 2001; Silla and Escudero, 2004). This definition and calculation of MRT, however, do not accord with the original definition of NUE as the ratio of dry mass production to N uptake (Hirose, 1971; Vitousek, 1982) when applied to non-steady-state systems. To avoid this difficulty, Hirose (2011) proposed defining MRT for N uptake: the expected length of time a unit N newly taken up from soil is retained in the plant body before being lost. It was calculated from plant N duration (time integral of standing plant N, g N d) divided by the amount of N taken up. Hirose (2012) applied this concept for the analysis of N use at leaf level, where leaf NUE was defined as the surplus production (i.e. carbon export from leaf = gross production minus leaf respiration; Monsi, 1960) per unit N allocated to leaf. Leaf NUE was factorized into leaf NP and MRT. Mean residence time of leaf N is the expected length of time a unit N newly allocated to the leaf is retained before being lost. Hirose and Oikawa (2012) extended this concept of MRT to cover leaf number, area and mass. MRT calculated for leaf number is the expected length of time a newly born leaf is retained until abscission, implying leaf longevity. Likewise, MRT of leaf area and dry mass is the expected retention time of newly produced leaf area and dry mass, respectively. Re-analysing leaf dynamics in the vegetative phase of *Xanthium canadense* grown at two N levels (Oikawa *et al*., 2005), they showed that all leaf variables had a higher MRT in the low than in the high N stand.

In this study, we investigate leaf dynamics from germination to full senescence of *X. canadense* plants grown at two stand densities. Two stands were established by planting one individual per pot with different spacing among pots so that plants at two densities received the same amount of soil resources but were grown in different light climates (Casper *et al*., 1998). To evaluate leaf dynamics in the context of plant development, we first analyse the effect of density on growth and reproduction. In the vegetative phase, dry mass will be allocated more to the main stem and less to leaves in a dense than in an open stand (e.g. Casper *et al*., 1998; for a meta-analysis, see Poorter *et al*., 2012*a*). Reproductive allocation may be higher in an open stand, because the higher branching activity will increase reproductive activities. Branching increases the number of meristems that potentially develop into reproductive shoots (Geber, 1990). Greater height growth in a dense stand may also occur at the expense of reproductive allocation.

If the light gradient in the stand accelerates senescence in lower older leaves (see above), leaf longevity will be shorter in a dense stand than in an open stand. However, we may alternatively hypothesize that leaf longevity will be shorter in the open stand because higher branching and reproductive activities are expected there. Both leaves and fruits of *X. canadense* have high N concentrations (Hirose *et al*., 2005) and will work as a strong N sink during growth of the plant. The existence of an N sink accelerates leaf senescence (Stoddart and Thomas, 1982; Chapin and Moilanen, 1991; Pugnaire and Chapin, 1992; Fischer, 2007; Yasumura *et al*., 2007). Nitrogen uptake from soil is usually not sufficient to meet high N demand in developing organs, and the rest is supplied from N in storage or senescing organs (Hirose, 1971; Millard, 1988; Chapin *et al*., 1990; Ono *et al*., 1996; Possato *et al*., 2001; Wright and Westoby, 2003; Marty *et al*., 2009). Then we may expect shorter leaf longevity in the open stand. The aim of this study is first to test the above two alternative hypotheses on leaf longevity: is leaf longevity shorter in the dense stand or in the open stand?

Stand density may thus influence leaf dynamics directly through different light gradients and indirectly through effects on growth and reproduction. Conversely, however, leaf dynamics may influence growth and reproduction of the plant. The second aim of this study is to evaluate the effects of leaf dynamics on dry mass production. The contribution of leaf variables to surplus production will be quantified. We define ‘leaf productivity’ as a surplus production per unit leaf variable (g [leaf variable]^−1^ d^−1^), together with their MRT to calculate ‘leaf efficiency’ (g [leaf variable]^−1^; Hirose and Oikawa, 2012). We expect a higher surplus production in plants in the open stand. However, investment of leaf number, area, mass and N may be higher in the open stand too. Then the efficiency as a ratio of the surplus production to the investment of leaf variables becomes a question: is the efficiency of investing a unit leaf variable also higher in the open stand? Efficiency of leaf area, dry mass, and N implies a leaf lifetime carbon gain (Oikawa *et al*., 2006), lifetime carbon return (Falster *et al*., 2011) and leaf-level NUE (Hirose, 2012), respectively. Productivity of leaf area implies ULR (unit leaf rate; Evans, 1972), the inverse of leaf mass productivity implies payback time of leaf construction (Saeki and Nomoto, 1958; Poorter, 1994), and leaf N productivity implies photosynthetic NUE (Field and Mooney, 1986).

## MATERIALS AND METHODS

### The experiment

An annual, *Xanthium canadense* (Asteraceae), was used for the experiment. Relatively large seed size allows for rapid growth to make a dense monospecific stand outcompeting other species in disturbed, nutrient-rich areas (Shitaka and Hirose, 1993). Fruits (burs) were collected from a large population at the shore of Lake Kamahusa (38°12'N, 140°41'E). Of two dimorphic seeds in a fruit, we used the lower seed that has no innate dormancy and a high germination potential (Esashi and Leopold, 1968).

The experiment was conducted in a shade house (5 × 10 × 2·5 m) made of steel frames covered with a sheet of white fly net, constructed in the experimental garden of Tokyo University of Agriculture, Tokyo (35°38'N, 139°38'E). The irradiance at ground level in the house was approx. 70 % of full sunlight. Monthly mean screen air temperature in the experimental period (2011) was 22·8 °C (June), 27·3 °C (July), 27·5 °C (August), 25·1 °C (September), 19·5 °C (October) and 14·9 °C (November). A total of 164 pots (1·5 L) filled with washed river sand were prepared. The pot size (1·5 L) might be small, but individuals received the same amount of soil resources including nutrients that were given throughout the experiment to minimize the pot size problem (Poorter *et al*., 2012*b*). Plant growth without root competition might be different from growth with root competition (Gersani *et al*., 2001). However, the present study is primarily concerned with above-ground competition for light that may influence plant architecture and leaf dynamics.

Seedlings with unfolded cotyledons, germinated on 20 June, were transplanted to the pots on 23 June (one plant per pot). On 29 June, pots were divided into two groups. Sixty-four pots were used to make an open stand with a density of 6·25 plants m^−2^; the other 100 pots were used to make a dense stand with a density of 59·2 plants m^−2^. A 0·35 mL aliquot of commercial fertilizer HYPONeX^®^ (6 % N, 10 % P_2_O_5_, 5 % K_2_O and other nutrients contained proportionately) was added per pot every 10 d from 29 June until 12 October (105 d after transplanting). This averaged at an N addition rate of 2·5 mg N pot^−1^ d^−1^. The growth experiment was continued until the whole plants fully senesced (10 November, 137 d after transplanting). Plants were watered with tap water as needed. On 18 July, the sides of the dense stand were enclosed with shade-cloth of 10 % light transmission to reduce radiation from the sides of the stand. The top of the shade-cloth was moved, tracking the height increase. The central 64 pots in the dense stand were used for measurements, and these pots were rotated every 10 d to remove position effects. Pots in the open stand were also rotated.

### Dimensions, dry mass and nitrogen

Seven focal plants were randomly selected per stand and marked by a waterproof pen for subsequent non-destructive determinations of leaf number and leaf dimensions. As three focal plants in the dense stand were damaged in the experiment, the final sample size for the dense stand was four. Plant height and basal stem diameter of focal plants were recorded at about 2 week intervals from 30 June (7 d after transplanting). Plant height was defined as the length from the stem base to the stem tip, and measured by a ruler to the nearest 1 mm. Basal stem diameter was determined by digital calipers to the nearest 0·1 mm. A geometric mean of two orthogonal measurements was taken for diameter. The number and order of leaves that newly emerged and that died were also recorded. Leaf emergence was defined when the leaf reached 20 mm in length. A numbered tag was attached around the petiole to identify the time of leaf emergence. Leaf death was defined when >90 % of the surface turned brown. Lamina length was measured by a ruler to the nearest 1 mm.

To estimate leaf area, dry mass and N of the focal plants, four other plants were randomly selected for destructive harvest from each stand at about 4 week intervals: 12 July (19 d after transplanting), 10 August (48 d), 2 September (74 d) and 8 October (105 d). After pots were removed, the remaining pots were rearranged to maintain the original density of the stands. On 10 November (137 d) focal plants were harvested. Plant height, basal stem diameter and lamina length of all leaves were determined in the same manner as for the focal plants. Harvested plants were separated into leaves, stems, roots and the reproductive part. At the final harvest (10 November), fruits were dissected into seeds and capsules. All leaves were identified by their position in the plant and determined individually. Leaf area was measured with image-processing software (Image J 1.44o, National Institute of Health, USA) after scanning (GT-9300UF, Epson, Nagano, Japan). The dry mass of the focal plants including roots but excluding leaves was estimated from regression:

$$M=a_{\text{M}}+b_{\text{M}}\times \text{log}\left(D^{\text{2}}\times H\right)$$

where *M* is dry mass, *D* basal stem diameter, *H* plant height, and *a*_M_ and *b*_M_ are constants, determined on every harvest occasion for samples from the open and the dense stand (*r*^2 ^= 0·73–0·98, open stand; *r*^2 ^= 0·13–0·91, dense stand). The lamina area of focal plants was estimated from regression:

$$A=a_{\text{A}}\times L^{b_{\text{A}}}$$

where *A* is lamina area, *L* lamina length, and *a*_A_ and *b*_A_ are constants, determined on every harvest occasion (*r*^2 ^= 0·76–0·98, open and dense stands combined). Sand particles were carefully removed from roots in running water. Dead leaves of focal plants produced between harvests were collected throughout the experiment. Dry mass was determined after oven drying at 60 °C to a constant weight. Nitrogen was determined with an NC-analyzer (Sumigraph NC-22F, Sumika-Bunseki, Osaka, Japan). Leaf mass per area (LMA, g m^−2^) and leaf N per area (LNA, g N m^−2^) were calculated and used to estimate leaf mass and N of focal plants, respectively.

### Measurements of respiration

Dark respiration of leaf, stem, root and the reproductive part was measured on every harvest occasion to determine surplus production of leaf. Measurements were done according to Kinugasa *et al*. (2005). Before measurement, plants were placed in the dark for 2–3 h for acclimation. All or a portion (depending on the volume) of plant materials was enclosed in an acrylic cylinder (119 mm in diameter and 82 mm in length, 0·91 L) connected to an open infrared gas analyser system (LI-6400; LICOR Inc., Lincoln, NE, USA). Materials were cut as needed to include them in the cylinder. An increase in respiration with cutting was disregarded because an earlier experiment with *X. canadense* showed that the increase was small (<3·5 %; Kinugasa *et al*., 2005). Measurement temperature was 28·0 °C (July), 27·0 °C (August), 25·5 °C (September), 22·0 °C (October) and 19·1 °C (November). The CO_2_ concentration in the cylinder was maintained at 380·0 ± 4·0 ppm (mean ± s.d.) and relative humidity at 70·2 ± 10·9 %. To estimate respiration in the growing season, *Q*_10_ tabulated in Kinugasa *et al*. (2005) was used, with daily mean air temperature recorded in Tokyo by the Japan Meteorological Agency (http://www.jma.go.jp/jma/index.html). They determined *Q*_10_ of leaf, stem, root and the reproductive part from temperature response curves, taken every 2 weeks by fitting for the respiration rates measured in the range 10–35 °C.

Surplus production was calculated from dry mass increase plus respiration of non-photosynthetic organs (gross production – leaf respiration = plant net production + heterotrophic respiration). To obtain surplus production in dry mass, the equivalence 1 mol of CO_2_ = 30 g of plant dry mass was assumed.

### Plant nitrogen use efficiency

Calculation and analysis of NUE followed Watari *et al*. (2012). NUE is the ratio of dry mass production (g) to N uptake (g N):

(1) NUE=ΔW/ΔN

where Δ*W* and Δ*N* include dry mass and N lost in the period Δ*t* as well as the increase in standing dry mass and N, respectively. NUE is factorized into NP and MRT (Hirose, 2011):

(2) $$\begin{array}{l} \Delta W/\Delta N=\left(\text{1}/N\right)\times \Delta W/\Delta t\times (N\times \Delta t/\Delta N)\\ \;\text{NUE}=\text{NP}\times \text{MRT} \end{array}$$

where *N* is the mean standing plant N. Nitrogen productivity is further factorized into leaf N productivity (LNP, g g^−1^ N d^−1^) and leaf N ratio (LNR, g N g^−1^ N):

(3) $$\begin{array}{l} \left(\text{1}/N\right)\Delta W/\Delta t=\left(\text{1}/N_{\text{L}}\right)(\Delta W/\Delta t)\cdot \left(N_{\text{L}}/N\right)\\ \text{NP}=\text{LNP}\times \text{LNR} \end{array}$$

where *N*_L_ denotes the standing amount of leaf N. Mean residence time is analysed into the ratio of plant N (PN, g N) to N uptake rate (NUR, g N d^−1^):

(4) $$\begin{array}{l} N\times \Delta t/\Delta N=N/(\Delta N/\Delta t)\\ \text{MRT}=\text{PN}/\text{NUR} \end{array}$$

Abbreviations and symbols used in this paper are listed in the Appendix.

### Leaf dynamics

The concept of MRT (Hirose, 2011, 2012) was employed for the analysis of leaf dynamics. Definition and calculation for MRT and related variables of leaf number, area, mass and N followed Hirose and Oikawa (2012). First, leaf duration (LD_i_, [i]d) in the period [0, *T*] is calculated:

(5) $${\text{LD}}_{\text{i}}=\int_{0}^{T}[f_{\text{i}}\left(t\right)–g_{\text{i}}\left(t\right)]\text{d}t$$

where *f*_i_(*t*) and *g*_i_(*t*) are the leaf production and loss at time *t*, respectively, both accumulated from the date of transplanting (*t* = 0) when no true leaf emerged. Subscript ‘i’ represents either one of the leaf variables: leaf number, leaf area, leaf mass or leaf N. When i = leaf number or area, ‘loss’ is equal to litter (dead leaf) production. When i = dry mass or N, ‘loss’ includes resorption as well as litter production. The standing mean of leaf variable-i (SL_i_, [i]) is given by

(6) $${\text{SL}}_{\text{i}}={\text{LD}}_{\text{i}}/\Delta T$$

where Δ*T* is the length of the leaf growth period. The MRT of leaf variable-i (MRT_i_) is given by

(7) $${\text{MRT}}_{\text{i}}={\text{LD}}_{\text{i}}/f_{\text{i}}\left(T\right)$$

The mean leaf productivity (LP_i_, g [i]^−1^ d^−1^) is given by

(8) $${\text{LP}}_{\text{i}}=P_{\text{s}}/{\text{LD}}_{\text{i}}$$

where *P*_s_ is the surplus production (g). Leaf efficiency (LE_i_, g [i] ^−1^) is the surplus production divided by the total investment of leaf variable-i, or the leaf productivity multiplied by leaf MRT:

(9) $${\text{LE}}_{\text{i}}=P_{\text{s}}/f_{\text{i}}\left(T\right)={\text{LP}}_{\text{i}}\times {\text{MRT}}_{\text{i}}$$

### Statistics

All growth variables were determined individually for focal plants (*n* = 7 in the open stand and *n* = 4 in the dense stand) and their mean and s.d. were calculated. Significance of the effects of stand density on the variables was tested by *t*-test. Effects of stand and age on growth variables were tested by two-way split-plot analysis of variance (ANOVA), with stand density and plant age as fixed effects. Differences in MRT among leaf variables were tested by two-way split-plot ANOVA, with stand density and leaf variable as fixed effects. Calculations were done with R (R Development Core Team, 2010).

## RESULTS

### Plant growth and reproduction

**Fig. 1.**
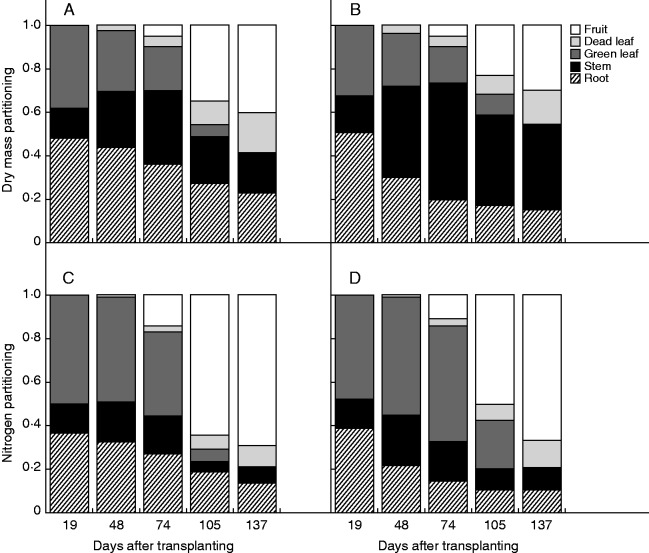
Partitioning of dry mass (A, B) and nitrogen (C, D) among organs in *Xanthium canadense* plants grown in an open (6·25 plants m^−2^; A, C) or in a dense stand (59·2 plants m^−2^; B, D) presented against days after transplanting (23 June). From the bottom: root (hatched), stem (black), green leaf (dark grey), dead leaf (light grey) and the reproductive part (light).

Plants in the open stand branched extensively and reached a maximal height of approx. 0·69 m, while in the dense stand plants reached 1·64 m with less branching. The dry mass ratio of branch shoot to main-stem shoot when the plant attained the maximal dry mass at 105 d after transplanting was 0·187 ± 0·067 (mean ± s.d.) in the open stand and 0·015 ± 0·004 in the dense stand. Thus, stand density strongly influenced plant form. However, dry mass production was only marginally higher in the open stand (Table 1). Nitrogen uptake showed no significant difference between the stands. The branch to main shoot N ratio (105 d) was 0·409 ± 0·133 in the open stand and 0·045 ± 0·014 in the dense stand. Higher ratios in N than dry mass indicate that branches worked as a strong N sink in plant growth and that the strength was higher in the open stand. Dry mass was allocated more to root in the open stand, while more was allocated to stems in the dense stand (Fig. 1). As compared with the open stand, N was allocated more to leaf and stem and less to root in the dense stand.

**Table 1.** Dry mass (DM) production, nitrogen (N) uptake, N use efficiency (NUE), N productivity (NP), mean residence time of plant N (MRT), leaf N productivity (LNP), leaf N ratio (LNR), plant N (PN) and N uptake rate (NUR) of *Xanthium canadense* plants grown in an open (6·25 plants m^−2^) or in a dense (59·2 plants m^−2^) stand

Variable^a^		Open	Dense
DM production	g per plant	28·3 (3·6)^b^	22·0 (7·7)^+^
N uptake	g N per plant	0·337 (0·021)	0·300 (0·049)^ns^
NUE	g g^−1^ N	84·5 (10·2)	72·2 (15·4)^ns^
NP	g g^−1^ N d^−1^	1·18 (0·13)	0·92 (0·23)*
MRT	d	71·4 (2·8)	79·5 (3·8)**
LNP	g g^−1^ N d^−1^	5·80 (0·44)	2·74 (0·63)***
LNR	–	0·204 (0·015)	0·333 (0·027)***
PN	g N per plant	0·174 (0·006)	0·171 (0·020)^ns^
NUR	mg N d^−1^ per plant	2·44 (0·16)	2·16 (0·36)^ns^

Significance between stands (*t*-test): ***P < 0·001, **P < 0·01, *P < 0·05, ^+^*P* < 0·1, ^ns^ *P* ≥ 0·1.

^a^DM production/N uptake = NUE = NP × MRT, NP = LNP × LNR, MRT = PN/NUR.

^b^Values are given the mean with s.d. in parentheses.

Reproductive allocation of dry mass was higher in the open stand (40 % vs. 30 %; Table 2). Reproductive yield (fruit mass) was nearly twice as high in the open stand as in the dense stand. Both fruit number and single fruit mass were higher in the open stand, though the difference was not significant owing to large variations among individuals. Reproductive N allocation was also higher in the open stand, although the difference was small (69 % vs. 67 %; Table 3). Higher allocation of N than dry mass to the reproductive part implies that reproduction also worked as a strong N sink in growth of the plant. Actually seeds in the fruits had a high N concentration (5·68 and 7·55 % in the open and dense stand, respectively; Supplementary Data Table S1), though the N concentration of capsules was extremely low (0·26 and 0·57 %, respectively). Of reproductive N, 24 % was remobilized from vegetative organs in the open stand (Table 3). The corresponding value was even higher, 47 %, in the dense stand.

**Table 2.** Reproductive allocation (= fraction of plant dry mass allocated for fruit production), reproductive mass (fruit dry mass), fruit number and single fruit dry mass of *Xanthium canadense* plants grown in an open (6·25 plants m^−2^) or in a dense (59·2 plants m^−2^) stand

Variable		Open	Dense
Reproductive allocation	–	0·402 (0·023)^a^	0·298 (0·039)***
Reproductive mass	g per plant	11·4 (1·5)	6·7 (3·2)**
Fruit number	Per plant	111 (50)	90 (70)^ns^
Single fruit mass	g per fruit	0·113 (0·029)	0·089 (0·030)^ns^

Significance between stands (*t*-test): ***P < 0·001, **P < 0·01, *P < 0·05, ^+^*P* < 0·1, ^ns^*P* ≥ 0·1.

^a^Values are given the mean with s.d. in parentheses.

**Table 3.** Reproductive nitrogen (N) allocation (= fraction of plant N allocated for fruit production), reproductive N and the amount of reproductive N that was retranslocated from leaf and stem plus root and that was taken up from soil, in *Xanthium canadense* plants grown in an open (6·25 plants m^−2^) or in a dense (59·2 plants m^−2^) stand

Variable		Open	Dense
Reproductive N allocation	–	0·693 (0·010)^a^	0·672 (0·015)*
Reproductive N	mg N per plant	234 (16)	202 (33)^+^
N retranslocated from leaf	mg N per plant	44 (4)	85 (13)***
stem + root	mg N per plant	12 (4)	10 (8)^ns^
N uptake from soil	mg N per plant	178 (18)	107 (27)***

Significance between stands (*t*-test): ***P < 0·001, **P < 0·01, *P < 0·05, ^+^*P* < 0·1, ^ns^*P* ≥ 0·1.

^a^Values are given the mean with s.d. in parentheses.

### Plant nitrogen use efficiency

Although dry mass production was higher in the open stand, no significant difference was observed in NUE between the stands (Table 1). A significant difference was found in the two components of NUE [eqn (2)]; NP was higher in the open stand, while MRT was higher in the dense stand. Higher NP in the open stand was expected due to a higher light availability there. However, lower MRT in the open stand was not expected. If leaves were maintained longer under less shady conditions, MRT would have been higher. Then, higher branching and reproductive activities in the open stand might have caused the lower MRT (see the Introduction).

Since the fraction of N allocated to leaves (LNR) was lower, higher NP in the open stand was due to the higher LNP [eqn (3)]. Significantly higher MRT in the dense stand resulted from the lower NUR for a slightly lower PN [eqn (4)], although the differences in both NUR and PN were not significant (Table 1).

### Leaf number

**Fig. 2.**
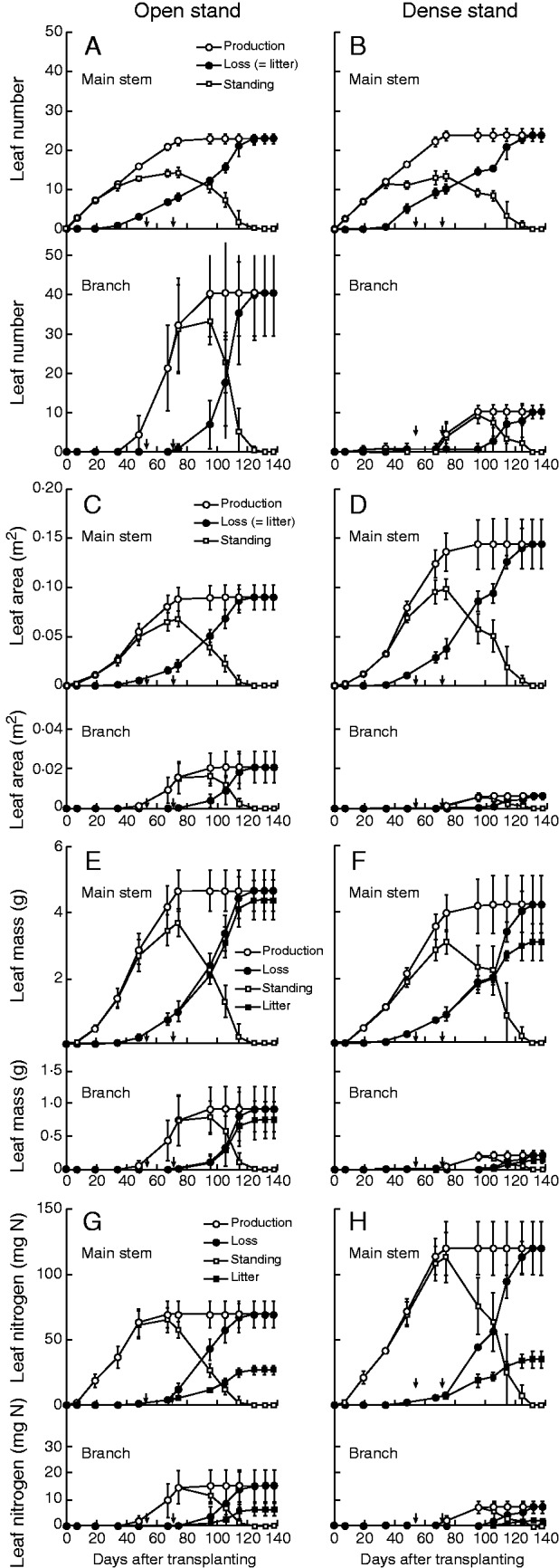
Leaf number (A, B), leaf area (C, D), leaf mass (E, F) and leaf nitrogen (G, H) on the main stem (upper panel) and on branches (lower panel) of *Xanthium canadense* plants grown in an open (6·25 plants m^−2^; A, C, E, G) or in a dense stand (59·2 plants m^−2^; B, D, F, H). In each panel, cumulative leaf production [*f*_i_ (*t*), open circles], cumulative loss [*g*_i_ (*t*), filled circles], the standing amount (open squares) and cumulative litter production (filled squares) are presented against days after transplanting (23 June). The difference between filled circles and filled squares represents ‘resorption’ (relevant only for leaf mass and nitrogen). Error bars denote ± 1 s.d. Arrows on the abscissa denote the date of flower bud formation (left) and of anthesis (right) first observed.

The number of leaves on the main stem increased linearly from transplanting until the onset of flowering at around 70 d (Fig. 2A, B; ANOVA, Supplementary Data Table S2). Leaf shedding began at around 40 d. As the slope of the curve was shallower than that of leaf production in the open stand, standing number increased, reaching a maximum at flowering time. Leaf shedding accelerated when fruit was growing, and standing leaf number decreased to zero by the end of the life of the plant. The pattern of leaf production on the main stem in the dense stand did not differ much from that in the open stands. However, as leaf shedding increased in parallel to leaf production in the dense stand, the maximal standing leaf number was smaller, was reached earlier and was maintained for longer (approx. 40 d) than in the open stand. In the open stand, the first branch leaves appeared at the time when the lowest leaves were shed. The number of branch leaves increased sharply until flowering, when the shedding of branch leaves was first observed. In the dense stand, production of branch leaves started later, at flowering time, and the maximum number was much smaller than in the open stand. It seems that branching induced shedding of leaves on the main stem in the open stand and that flowering induced branching in the dense stand.

Total production of leaf number, duration and standing mean of main-stem leaves were not different between the stands, but longevity (= MRT of leaf number) of main-stem leaves was higher in the open stand: 49·2 vs. 45·7 d (Table 4). For branch leaves, production, duration, standing mean and longevity were all higher in the open stand, though longevity (38·9 vs. 32·9 d) was not significant owing to large variations among individuals.

**Table 4.** Total leaf production, litter production, leaf duration, standing mean and mean residence time of leaf number, leaf area, leaf mass and leaf nitrogen of main-stem and branch leaves in *Xanthium candense* plants grown in an open (6·25 plants m^−2^) or in a dense (59·2 plants m^−2^) stand

Variable	Leaf number		Leaf area		Leaf mass		Leaf nitrogen	
	Open	Dense	Open	Dense	Open	Dense	Open	Dense
Leaf production	per plant		m^2^ per plant		g per plant		mg N per plant	
Main stem	22·9 (1·2)	24·0 (1·6)^ns^	0·090 (0·013)	0·144 (0·026)**	4·67 (0·63)	4·23 (0·88)^ns^	69·2 (10·1)	119·6 (20·4)***
Branch	40·4 (10·9)	10·3 (1·7)***	0·021 (0·008)	0·006 (0·002)**	0·91 (0·34)	0·21 (0·07)**	15·1 (6·0)	7·1 (2·2)*
Litter production	per plant		m^2^ per plant		g per plant		mg N per plant	
Main stem	22·9 (1·2)	24·0 (1·6)^ns^	0·090 (0·013)	0·144 (0·026)**	4·38 (0·61)	3·08 (0·56)**	26·9 (3·6)	34·9 (6·6)*
Branch	40·4 (10·9)	10·3 (1·7)***	0·021 (0·008)	0·006 (0·002)**	0·75 (0·28)	0·14 (0·04)**	6·3 (2·4)	2·0 (0·6)**
Leaf duration	d		m^2^ d		g d		g N d	
Main stem	1124 (74)	1095 (60)^ns^	4·00 (0·64)	5·97 (1·02)**	217 (36)	201 (40)^ns^	4·02 (0·62)	7·08 (1·46)***
Branch	1572 (446)	339 (154)***	0·75 (0·30)	0·20 (0·10)**	36 (14)	7 (4)**	0·62 (0·25)	0·24 (0·12)*
Standing mean	per plant		m^2^ per plant		g per plant		g N per plant	
Main stem	9·2 (0·7)	9·0 (0·5)^ns^	0·033 (0·006)	0·048 (0·005)**	1·78 (0·32)	1·63 (0·20)^ns^	0·033 (0·006)	0·057 (0·007)***
Branch	12·9 (3·7)	2·7 (1·1)***	0·006 (0·002)	0·002 (0·001)**	0·29 (0·11)	0·05 (0·02)**	0·005 (0·002)	0·002 (0·001)*
Mean residence time	d		d		d		d	
Main stem	49·2 (2·5)	45·7 (2·2)*	44·3 (1·5)	41·5 (1·4)*	46·4 (2·7)	47·5 (2·0)^ns^	58·1 (3·0)	58·9 (2·2)^ns^
Branch	38·9 (2·3)	32·6 (12·3)^ns^	35·7 (1·6)	30·6 (9·3)^ns^	38·9 (1·3)	30·5 (7·7)*	40·7 (2·5)	32·3 (9·9)^+^

Leaf growth period: 122 ± 6 (mean ± s.d.) d in the open stand, and 123 ± 10 d in the dense stand.

Note that litter production is the same in amount as leaf production in leaf number and area, while it is lower in leaf mass and nitrogen due to resorption. See Fig. 2.

Significance between stands (*t*-test): ***P < 0·001, **P < 0·01, *P < 0·05, ^+^*P* < 0·1, ^ns^*P* ≥ 0·1.

^a^Values are given the mean with s.d. in parentheses.

### Leaf area

Because the size of individual leaves was smaller on branches (Table 5), leaf area production was much lower on branches than on the main stem (Fig. 2C, D). As individual leaf area was larger in the dense stand, total production of leaf area and the peak standing leaf area were higher. Peak standing leaf area was observed at flowering time in both stands, when LAI (leaf area per ground area) was calculated as 0·43 in the open stand and 5·8 in the dense stand. Leaf area duration and standing mean leaf area were higher in the dense stand than in the open stand, and on the main stem than on branches (Table 4). Mean residence time of leaf area was longer in the open stand (44·3 vs. 41·5 d on the main stem, and 35·7 vs. 30·6 d on branches), though the difference for branch leaves was not significant.

**Table 5.** Single leaf area and leaf mass, leaf mass per area (LMA), leaf nitrogen per area (LNA) and leaf nitrogen concentration (LNC) of main-stem and branch leaves in *Xanthium canadense* plants grown in an open (6·25 plants m^−2^) or in a dense (59·2 plants m^−2^) stand

Variable^a^			Open	Dense
Leaf area	10^−4 ^m^2^ per leaf	Main stem	35·4 (4·4)^b^	54·4 (7·5)***
		Branch	4·64 (0·74)	6·04 (2·55)^ns^
Leaf mass	g per leaf	Main stem	0·192 (0·025)	0·183 (0·030)^ns^
		Branch	0·022 (0·004)	0·021 (0·009)^ns^
LMA	g m^−2^	Main stem	54·3 (0·5)	33·6 (1·4)**
		Branch	47·9 (0·3)	34·0 (0·7)***
LNA	g N m^−2^	Main stem	1·01 (0·05)	1·18 (0·11)**
		Branch	0·83 (0·05)	1·19 (0·04)***
LNC	mg N g^−1^	Main stem	18·6 (1·0)	35·3 (2·2)***
		Branch	17·2 (1·0)	35·0 (1·4)***

^a^Calculated from leaf duration (Table 4): leaf area = LD_area_/LD_number_, leaf mass = LD_mass_/LD_number_, LMA = LD_mass_/LD_area_, LNA = LD_nitrogen_/LD_area_, LNC = LD_nitrogen_/LD_mass._

^b^Values are given the mean with s.d. in parentheses.

### Leaf mass

In contrast to leaf area, leaf mass production on the main stem did not differ between the stands (Fig. 2E, F) due to the higher LMA in the open stand (Table 5). Because part of the dry mass was resorbed from old leaves before shedding, total litter production was smaller than the total leaf production (Table 4). The difference between leaf production and litter production was larger in the dense stand. Dry mass resorption was calculated from this difference [= (leaf produciton – litter production)/leaf production; Table 6], disregarding leaching and respiratory loss (i.e. the maximum value of cumulative leaf production once attained was maintained toward the end of a plant’s life). It was significantly lower in the open stand (6 % vs. 27 %, main-stem leaves; 18 % vs. 34 %, branch leaves). In both stands, resorption efficiency was higher in branch leaves. No significant difference was found in leaf duration, standing mean and MRT on the main stem between stands. However, on branches they were all significantly higher in the open stand.

**Table 6.** Dry mass and nitrogen resorption in main-stem and branch leaves, calculated as the difference between leaf production and litter production for *Xanthium canadense* plants grown in an open (6·25 plants m^−2^) or in a dense (59·2 plants m^−2^) stand

Variable^a^	Leaves	Open	Dense	
DM resorption (g per plant)	Main stem	0·28 (0·09)^b^	1·15 (0·35)	***
	Branch	0·17 (0·06)	0·07 (0·03)	*
DM resorption efficiency (–)	Main stem	0·06 (0·02)	0·27 (0·03)	***
	Branch	0·18 (0·03)	0·34 (0·05)	***
N resorption (mg N per plant)	Main stem	42·3 (7·2)	84·7 (13·9)	***
	Branch	8·8 (3·8)	5·1 (1·7)	ns
DM resorption efficiency (–)	Main stem	0·61 (0·03)	0·71 (0·01)	***
	Branch	0·58 (0·04)	0·71 (0·02)	***
N/DM resorption ratio (g N g^–1^)	Main stem	0·158 (0·042)	0·076 (0·010)	**
	Branch	0·054 (0·016)	0·072 (0·012)	+

Significance between stands (*t*-test): ***P < 0·001, **P < 0·01, *P < 0·05, ^+^*P* < 0·1, ^ns^*P* ≥ 0·1.

^a^Resorption = leaf production – litter production, see Table 4 for leaf production and litter production, Resorption efficiency = resorption/leaf production, N/DM resorption ratio = N resorption/DM resorption.

^b^Values are given as the mean with the s.d. in parentheses.

### Leaf nitrogen

The total amount of N allocated to main-stem leaves was nearly twice as high in the dense stand (Fig. 2G, H; Table 4), reflecting on a higher leaf N concentration in the dense stand (Table 5). Note that this higher LNC caused higher LNA in the dense stand (Table 5), contrary to the expectation that LNA would be lower in the dense stand where light availability was limited (see the Introduction). This may be because a limited availability of carbon did not allow for further expansion of leaf area to use N efficiently (Hirose, 1984) even though the fraction of N allocated to leaf increased (Table 1).

The amount of N in litter was much smaller than the N allocated to leaves, since a large part of the leaf N was resorbed before shedding. The resorption efficiency (calculated assuming no leaching loss) of leaves on the main stem was 61 % in the open stand and 71 % in the dense stand (Table 6). Corresponding values on branches were 58 and 71 %, respectively. Although leaf N duration on the main stem was higher in the dense stand, MRT of leaf N did not differ between the two stands because leaf N allocation was also higher in the dense stand. Mean residence time was 58 and 59 d in the open and the dense stand, respectively. These values were higher than those for other leaf variables that were in the range of 41–49 d (Table 4; ANOVA, Supplementary Data Table S3). High MRT in leaf N is attributable to the substantial resorption before leaf shedding.

### Life expectancy of individual leaves

Life expectancy, or retention time of leaf cohorts (that was identified by emergence time and calculated from data for Fig. 2), is presented in Table 7. On the main stem, retention time increased to a maximum in the cohort that emerged around 50 d, and then decreased. Note that 50 d was the time at which flower buds were first observed (Fig. 2), but also note that leaf ‘emergence’ was defined as when leaf length reached 20 mm (see the Materials and Methods), about 3 (dense) or 4 d (open stand) after a leaf tip was first observed. In cohorts ‘emerged’ on the main stem before 50 d, retention time of leaf number, area and mass on the main stem was longer in the open than in the dense stand, whereas in cohorts emerged later, retention time was shorter in the open stand. However, N retention time of main-stem leaves tended to be longer in the dense stand throughout the life of the plant. Retention time of branch leaves was longer in the open stand, except for the latest cohort.

**Table 7.** Retention time (d) of leaf cohorts on the main stem and branches in *Xanthium canadense* plants grown in an open (6·25 plants m^−2^) or in a dense (59·2 plants m^−2^) stand

Emergence (d)	Leaf number		Leaf area		Leaf mass		Leaf nitrogen	
	Open	Dense	Open	Dense	Open	Dense	Open	Dense
Main stem								
10	42·4	33·6	35·8	28·4	36·2	30·7	58·6	61·8
20	50·9	38·4	41·4	31·3	40·3	38·2	59·5	62·2
30	55·6	46·7	45·0	35·5	46·1	44·9	57·6	60·1
40	58·0	52·1	46·8	35·3	50·0	48·4	58·8	63·8
50	56·2	57·7	49·0	44·4	51·4	56·7	60·6	59·6
60	50·6	52·8	46·5	48·8	47·7	51·5	54·1	55·8
70	46·7	53·8	42·5	46·8	43·5	49·7	50·0	57·6
Branch								
40	37·6		35·6		36·8		37·7	
50	43·0		36·8		41·5		40·1	
60	42·6		40·1		42·7		45·1	
70	39·1	33·5	37·8	32·6	38·9	34·7	41·9	31·3
80	33·2	32·0	32·0	31·1	33·1	30·2	41·1	31·0
90	30·4	36·8	26·4	34·9	28·7	34·5	33·1	36·5

Cohorts are defined by leaf emergence time in days after transplanting (23 June)

Retention time of cohort *t* is calculated at Δ*t* that satisfies *f*_i_ (*t*) = *g*_i_ (*t* + Δ*t*) (see Fig. 2)

### Surplus production and leaf efficiency

Surplus production calculated from dark respiration (Supplementary Data Table S4) and dry mass increase (Table 1) was 53·1 and 38·4 g per plant in the open and in the dense stand, respectively (Table 8). These amounts were exported for construction of new tissues elsewhere and maintenance of heterotrophic tissues in the plant. Of surplus production, 53 and 57 % was converted to dry mass in the plant (28·3 and 22·0 g per plant) in the open and the dense stand, respectively. Since surplus production is the outcome of invested leaf number, area, dry mass and N, leaf efficiency defined as surplus production per unit investment of these leaf variables should be an important parameter of leaf activity in the canopy. Leaf efficiency for leaf number was 0·87 and 1·12 g per leaf in the open and the dense stand, respectively. These values imply a lifetime carbon gain of a single leaf in the respective stands. Lower gain in the open stand resulted simply from a smaller leaf size in this stand (Table 5). When evaluated in terms of leaf area, the open stand had a higher lifetime gain: 479 vs. 254 g m^−2^. In leaf mass, the open stand still had a higher efficiency, but the difference was marginal. The values (9·5 and 8·6 g g^−1^) imply the lifetime carbon return relative to leaf mass; i.e. a leaf produced dry mass around nine times larger than its own mass in life. The difference in leaf efficiency between stands was greatest in terms of N (633 vs. 302 g g^−1^ N). Leaf N produced dry mass more than twice as efficiently in the open stand.

**Table 8.** Surplus production (= carbon export from leaf), total investment (= sum of leaf production on the main stem and branches in Table 4), leaf productivity, mean residence time, and efficiency of leaf number, area, dry mass and nitrogen of *Xanthium canadense* plants grown in an open (6·25 plants m^−2^) or in a dense (59·2 plants m^−2^) stand

Variable	Leaf number		Leaf area		Leaf mass		Leaf nitrogen	
	Open	Dense	Open	Dense	Open	Dense	Open	Dense
Surplus production	g per plant		g per plant		g per plant		g per plant	
	53·1 (5·4)^a^	38·4 (9·6)**	53·1 (5·4)	38·4 (9·6)**	53·1 (5·4)	38·4 (9·6)**	53·1 (5·4)	38·4 (9·6)**
Total investment	Per plant		m^2^ per plant		g per plant		g N per plant	
	63·3 (11·5)	34·3 (1·7)***	0·111 (0·010)	0·150 (0·027)**	5·58 (0·49)	4·45 (0·94)*	0·084 (0·009)	0·127 (0·022)**
Leaf productivity	mg leaf ^−1^ d^−1^		g m^−2^ d^−1^		mg g^−1^ d^−1^		g g^−1^ N d^−1^	
	20·3 (4·8)	26·6 (4·4)^+^	11·2 (0·6)	6·2 (0·5)***	211 (12)	184 (13)**	11·5 (0·8)	5·2 (0·6)***
Mean residence time	d		d		d		d	
	42·8 (2·3)	41·8 (4·7)^ns^	42·7 (1·8)	41·1 (1·6)^ns^	45·2 (2·4)	46·7 (2·2)^ns^	55·0 (3·5)	57·5 (2·3)^ns^
Leaf efficiency	g per leaf		g m^−2^		g g^−1^		g g^−1^ N	
	0·87 (0·24)	1·12 (0·27)^ns^	479 (33)	254 (26)***	9·52 (0·61)	8·62 (0·96)^+^	633 (70)	302 (42)***

Significance between stands (*t*-test): ***P < 0·001, **P < 0·01, *P < 0·05, ^+^*P* < 0·1, ^ns^*P* ≥ 0·1.

^a^Values are given the mean with s.d. in parentheses.

Leaf efficiency was factorized into LP and MRT [eqn (9)]. Leaf productivity is the surplus production per unit standing leaf variable per unit time [eqn (8)]. It was higher in the open stand for all leaf variables except leaf number (Table 8). Payback time was calculated from LP_mass_ as 6·6 and 7·6 d in the open and in the dense stand, respectively [multiplied by a factor of 1·4 according to Poorter (1994)]. The biomass invested for leaf construction was reimbursed in around 7 d, with 1 d earlier in the open than in the dense stand. In these calculations, leaves on the main stem and branches were combined. When combined, however, differences in MRT observed in Table 4 disappeared in all leaf variables (Table 8).

## DISCUSSION

### Leaf longevity and MRT

Plants were grown individually in pots without root competition, and size variability was limited, which was different from plants growing in the field (e.g. Nagashima *et al*., 1995). However, plant architecture mimicked the one observed in natural stands, where uncrowded plants have a shorter stature with more branching than crowded plants (e.g. Weiner *et al*., 1990). Low variation in plant size was indispensable for detailed evaluation of leaf dynamics. In this study we showed that stand density influenced plant architecture strongly, but changed dry mass production only marginally. Branching and reproductive activities were higher in the open stand. These activities indirectly affected leaf dynamics. We expected that leaves in the open stand would have a higher leaf longevity and MRT, because the leaves exposed to near-full light would maintain their activity longer (see the Introduction; but see also Osada, 2013). Light environment influences leaf birth, death and longevity. Bazzaz and Harper (1977) showed in flax that the leaf birth rate increased in less dense stands; however, as the death rate increased even more, life expectancy decreased. Shallower light gradients increased leaf longevity of a tropical pioneer tree (Ackerly and Bazzaz, 1995). Contrary to our expectation, we found no significant difference in leaf longevity and MRT between the two stands (Table 8). However, when leaves on the main stem and branches were distinguished (Table 4), main-stem leaves had a higher MRT in leaf number and leaf area in the open stand, but not in leaf mass and N. In contrast, branch leaves showed no significant difference in MRT in leaf number and leaf area, but significantly higher MRT in leaf mass and leaf N in the open stand.

When MRT was determined for every leaf cohort (Table 7), early cohorts always had a longer MRT in the open stand in all leaf variables except leaf N. They were less influenced by branching and reproductive activities. Later cohorts (emerged after flower budding) had a shorter MRT in the open stand. These results suggest that leaf longevity and MRT are higher when all leaves are exposed to near-full light *and* when branching and reproductive activities are low. In the present study, this was manifested in the early cohorts in the open stand. This conforms to earlier findings in other experimental works, where non-branching, non-reproductive plants were employed (Hirose *et al*., 1988; Schieving *et al*., 1992; Hikosaka *et al*., 1994; Ackerly and Bazzaz, 1995). Shorter MRT in plants of higher branching and reproductive activities was demonstrated in later cohorts in the open stand. Schmid and Bazzaz (1994) showed that leaf turnover rates (inverse of leaf longevity) were higher in a species having branched shoots (*Aster lanceolatus*) than in a species having non-branched shoots (*Solidago canadensis*). In the early vegetative phase, light environment strongly controlled leaf longevity, whereas in the later growth stage the effect of branching and reproductive activities over-ruled the effect of light environment in controlling leaf longevity. This was shown in the open stand where branching and reproductive activity were higher: here leaf longevity was reduced in the later stage.

### Nitrogen resorption

Many authors have suggested that the existence of an N sink accelerates N resorption when N uptake from soil is limited, and induces leaf senescence (see the Introduction). Excision of flower buds or growing apices reduced N resorption and delayed senescence in pre-existing leaves (Derman *et al*., 1978; Crafts-Brandner *et al*., 1987; Chapin and Moilanen, 1991; Karlsson, 1994; Marty *et al*., 2009; but see Hikosaka *et al*., 2010). In the present experiment, growing shoot tips worked as an N sink in the early vegetative phase, whereas in the later growth branching and fruit maturation worked as a stronger N sink. Particularly in annuals, most N in the vegetative body is remobilized to the reproductive part before plant death (Sinclair and de Wit, 1975; Hocking and Steer, 1983; Hirose *et al*., 2005). However, resorption is strongly controlled by phloem loading (Chapin and Moilanen, 1991). When flowering of *X. canadense* was delayed by 1 month by controlling photoperiod but otherwise grown in the natural environment, N resorption was retarded (phloem loading reduced) due to low temperatures, and leaves did not die until the first frost (Shitaka and Hirose, 1998). The observed N resorption efficiencies, 60 % in the open stand and 71 % in the dense stand, were in the range compiled by several authors (Aerts, 1996; Kobe *et al*., 2005; Vergutz *et al*., 2012). Yasumura *et al*. (2007) showed that N resorption was lower when sink activity was low, but did not increase when plants had a high N sink. However, different resorption efficiencies between the open and the dense stand did not seem to have resulted from different sink strength. Other factors might have been involved in the difference, e.g. difference in composition and degradability of leaf N (Yasumura *et al*., 2007), intensity of nutrient and water stress (Pugnaire and Chapin 1992), etc. There was a positive correlation between N resorption and LNA. Both N resorption and LNA were higher in the dense than in the open stand, and on branches than on the main stem, although many other studies did not find such correlations (Aerts, 1996). A meta-analysis of N resorption performed across species showed that N resorption tended to be low in leaves of high N concentrations (Kobe *et al*., 2005; Vergutz *et al*., 2012).

Nitrogen resorption is accompanied by dry mass resorption, because N is retranslocated in organic forms such as amino acids and amides, not in inorganic ions (Chapin and Kedrowski, 1983; Millard, 1988; Fischer, 2007). Efficiency of dry mass resorption, calculated disregarding leaching and respiratory loss, varied from 6 to 34 %. Smaller values but larger variations in dry mass than N resorption have been shown in other studies, and the present values were in the range presented in those studies (Chapin and Kedrowski, 1983; Vergutz *et al*., 2012). The ratio of N to dry mass resorption that may indicate the mean N concentration of the substance for translocation also varied considerably from 5·4 to 15·8 % (Table 6). A value of 15·8 % is closer to the value (19·2 %) for glutamine that Norby *et al*. (1990) assumed as a translocating substance in tree leaves.

### Nitrogen use efficiency

No significant difference was observed in plant NUE between the open and the dense stand, due probably to large variation among individuals, because earlier studies conducted in a similar experimental set-up showed a higher NUE in plants grown in an open than in a dense stand (Nishimura *et al.*, 2010; Watari *et al*., 2012). However, significant differences were observed in the components of NUE: NP was higher in the open stand and MRT was higher in the dense stand. A higher NP associated with a lower MRT, and vice versa, was first discussed in interspecific comparisons by Berendse and Aerts (1987). They suggested an evolutionary trade-off existing between NP and MRT (see also Aerts, 1990; Garnier and Aronson, 1998; Aerts and Chapin, 2000). This trade-off may reflect the negative correlation between photosynthetic capacity and leaf life span at leaf level (Wright *et al*., 2004). Vincent (2006) showed that tropical tree seedlings had lower photosynthetic capacities with longer life spans when grown in shady conditions. Trade-offs were thus observed in a phenotypic response to different light conditions (see Eckstein and Karlsson, 2001).

**Fig. 3.**
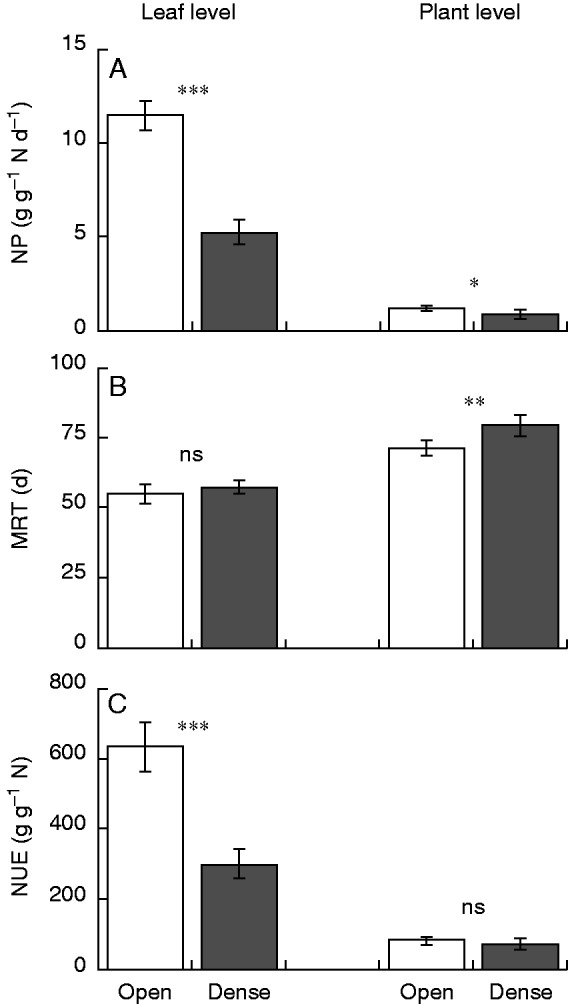
Leaf- and plant-level nitrogen productivity (NP; A), mean residence time (MRT; B) and nitrogen use efficiency (NUE; C) of *Xanthium canadense* plants grown in an open (6·25 plants m^−2^, white) or in a dense stand (59·2 plants m^−2^, grey). NP × MRT = NUE. Leaf-level variables are from Table 8; plant-level variables are from Table 1. Error bars denote ± 1 s.d. ***P < 0·001, **P < 0·01, *P < 0·05, ^+ ^*P* < 0·1, ^ns^*P* ≥ 0·1.

Nitrogen plays a crucial role in photosynthetic production (Evans, 1989; Hikosaka and Terashima, 1995). Most plant N is allocated to green leaves (e.g. Hocking and Steer, 1983; this study), and most leaf N to chloroplasts (Evans and Seemann, 1989; Makino *et al*., 2003). There is a correlation between N uptake and dry mass growth (e.g. Hirose, 1978) as well as between leaf N and photosynthetic capacity (Field and Mooney, 1986; Evans, 1989). Then leaf N use in photosynthetic production and plant N use in dry mass production both are important for understanding plant functioning in a given environment. Hirose (2011) re-analysed the data on N use in a perennial *Solidago altissima* and an annual *Amaranthus patulus* stand (Hirose, 1975), in which higher NUE in *S. altissima* than in *A. patulus* was explained by a higher MRT in the former. Extensive recycling of N between above- and below-ground parts as well as between new and old leaves accounted for the higher MRT of plant N in the perennial system. At leaf level, a higher NUE in *S. altissima* was also explained by a higher MRT of leaf N (Hirose, 2012). NUE and NP are higher at leaf level than at plant level because N allocation to leaves is a fraction of N taken up from soil, and net dry mass production is a fraction of surplus production. MRT is lower at leaf level than at plant level because leaf turnover is higher than plant turnover due to the N recycling between leaf and other structures at plant level. The same was observed in the present study: NUE and NP were higher, and MRT was lower at leaf than at plant level (Fig. 3). However, a difference in MRT between stands was observed at plant level, but was not observed at leaf level. In contrast, a difference in NUE between stands was observed at leaf level, but was not observed at plant level. A difference in NUE at leaf level between stands reflects the difference in NP more than the difference in MRT. It is intriguing that the effect of different light climates on NUE via NP is more evident at leaf level than at plant level. This may be because the light environment influences photosynthetic productivity directly, and N allocation indirectly within a plant. Lower NP at leaf level (closely related to LNP) in the dense stand was compensated for by a higher allocation of N to leaf (LNR) at the plant level (Table 1). Thus, only a small difference was found in NP at plant level between the two stands.

### Concluding remarks

Stand density influences leaf dynamics through photosynthetic activity and associated N allocation among leaves, and also through the effects on growth, form and reproduction of the plant. New leaves constitute a strong N sink for older leaves, branches for the main stem leaves, and reproduction for all leaves. They all induce remobilization of N and finally lead to senescence of the leaf. The effects of stand density on leaf birth, death and longevity were analysed well with the concept MRT applied to leaf number, area, mass and N. Nitrogen use is a key process to link leaf dynamics with plant growth and reproduction. We showed how leaf-level NUE was integrated into plant-level NUE with the analysis of components of NUE at respective levels. The concepts of efficiency, productivity and MRT applied at leaf and at plant level may provide a useful framework for clarifying plant functioning in the globally changing world.

## SUPPLEMENTARY DATA

Supplementary data are available online at www.aob.oxfordjournals.org and consist of the following. Table S1: dry mass and nitrogen of capsules and seeds of *X. canadense* plants. Table S2: analysis of variance for the effects of stand density and plant age on leaf number, leaf area, leaf mass and leaf nitrogen of *X. canadense* plants. Table S3: analysis of variance for the difference in mean residence time among leaf variables of *X. canadense* plants. Table S4: specific respiration rates of leaf, stem, root and the reproductive part in *X. canadense* plants.

Supplementary Data

## APPENDIX

List of growth variables

**Table:**

Growth variables	Explanation	Unit
*f*_i_ (*t*)	Cumulative production (investment) of leaf variable-i*	[i]^†^
*g*_i (_*t*)	Cumulative loss of leaf variable-i	[i]
LD_i_	Duration of leaf variable-i	[i] d
LE_i_	Photosynthetic efficiency of leaf variable-i	g [i]^−1^
LMA	Leaf mass per area	g m^−2^
LNA	Leaf nitrogen per area	g N m^−2^
LNP	Leaf nitrogen productivity	g g^−1^ N d^−1^
LNR	Leaf to plant nitrogen ratio	–
LP_i_	Photosynthetic productivity of leaf variable-i	g [i]^−1^ d^−1^
MRT	Mean residence time	d
NP	Nitrogen productivity	g g^−1^ N d^−1^
NUE	Nitrogen use efficiency	g g^−1^ N
NUR	Nitrogen uptake rate	g N d^−1^
*P*_s_	Surplus production	g
PN, *N*	Plant nitrogen	g N
SL_i_	Standing mean of leaf variable-i	[i]
*t*	Time from transplanting	d
*T*	Time at the end of growth	d
*W*	Plant dry mass	g

* i, either leaf number, leaf area, leaf mass or leaf nitrogen.

^†^[i], either none (leaf number), m^2^ (leaf area), g (leaf mass) or g N (leaf nitrogen).
