# Supplementary material for: Leaf dynamics in growth and reproduction of Xanthium canadense as influenced by stand density
Source: Ann Bot. 2015 Aug 5;116(5):807–19. doi: 10.1093/aob/mcv114 (PMC4590326; doi:10.1093/aob/mcv114)
Supplement: Supplementary Data [file supp_116_5_807__index.html]

Supplementary Data 

# Leaf dynamics in growth and reproduction of *Xanthium canadense* as influenced by stand density

## Supplementary Data

files

- Supplementary Data - pdf file
