# Supplementary material for: Leaf dynamics in growth and reproduction of Xanthium canadense as influenced by stand density
Source: Ann Bot. 2015 Aug 5;116(5):807–19. doi: 10.1093/aob/mcv114 (PMC4590326; doi:10.1093/aob/mcv114)
Supplement: Supplementary Data [file supp_mcv114_aob-15275-s01.pdf]

SUPPLEMENTARY DATA

Table S1. Dry mass and nitrogen of capsules and seeds of *Xanthium canadense* plants grown in an open (6.25 plants m^{-2}) or in a dense (59.2 plants m^{-2}) stand

	Dry mass (g $plant^{-1}$)		Nitrogen (mg $plant^{-1}$)		N concentration (%)	
	Open	Dense	Open	Dense	Open	Dense
Capsules	7.56 (1.78)[a]	4.50^{+} (3.37)	21 (18)	35^{ns} (49)	0.26 (0.15)	0.57^{+} (0.40)
Seeds	3.81 (0.78)	2.21** (0.53)	213 (31)	167^{+} (38)	5.68 (0.53)	7.55*** (0.28)
Total	11.4 (1.53)	6.7** (3.2)	234 (16)	202^{+} (33)	2.08 (0.26)	3.31** (0.90)

[a] Values are mean and SD in parentheses. Significance between stands (*t*-test): *** $P < 0.001$, ** $P < 0.01$, * $P < 0.05$, $^{+}$ $P < 0.1$, ^{ns} $P \geq 0.1$.

Table S2. Analyses of variance for the effects of stand density and plant age on leaf number, leaf area, leaf dry mass and leaf nitrogen of *Xanthium canadense* plants grown in an open (6.25 plants m^{-2}) or in a dense (59.2 plants m^{-2}) stand. See Fig. 2

Leaf variable		Stand	Age	Stand × Age
Main-stem leaves				
Leaf number	Standing amount	0.889[a]	**0.004**	0.814
	Cumulative production	0.115	< **0.001**	0.496
	Cumulative loss	0.054	< **0.001**	0.620
Leaf area	Standing amount	**0.004**	0.428	0.725
	Cumulative production	< **0.001**	< **0.001**	< **0.001**
	Cumulative loss	< **0.001**	< **0.001**	< **0.001**
Leaf dry mass	Standing amount	0.807	0.600	0.465
	Cumulative production	0.218	< **0.001**	0.327
	Cumulative loss	**0.006**	< **0.001**	< **0.001**
	Cumulative resorption	< **0.001**	< **0.001**	< **0.001**
Leaf nitrogen	Standing amount	< **0.001**	0.165	0.349
	Cumulative production	< **0.001**	< **0.001**	< **0.001**
	Cumulative loss	**0.020**	< **0.001**	< **0.001**
	Cumulative resorption	< **0.001**	< **0.001**	< **0.001**
Branch leaves				
Leaf number	Standing amount	< **0.001**	**0.045**	0.495
	Cumulative production	< **0.001**	< **0.001**	< **0.001**
	Cumulative loss	**0.002**	< **0.001**	< **0.001**
Leaf area	Standing amount	**0.007**	**0.031**	0.625
	Cumulative production	**0.007**	< **0.001**	< **0.001**
	Cumulative loss	**0.009**	< **0.001**	< **0.001**
Leaf dry mass	Standing amount	**0.003**	**0.038**	0.477
	Cumulative production	**0.004**	< **0.001**	< **0.001**
	Cumulative loss	**0.005**	< **0.001**	< **0.001**
	Cumulative resorption	**0.016**	< **0.001**	< **0.001**
Leaf nitrogen	Standing amount	**0.027**	0.066	0.843
	Cumulative production	**0.024**	< **0.001**	< **0.001**
	Cumulative loss	**0.010**	< **0.001**	< **0.001**
	Cumulative resorption	0.062	< **0.001**	< **0.001**

[a] *P*-values are presented; **bold** for $P < 0.05$.

Table S3. Analyses of variance for the difference in MRT among leaf variables of *X. canadense* plants grown in an open (6.25 plants m^{-2}) or in a dense (59.2 plants m^{-2}) stand (see Table 4 for MRT of main-stem and branch leaves and Table 8 for MRT for all leaves)

	Stand[a]	Variable[b]	Stand x variable
Main stem leaves	0.431[c]	**< 0.001**	**< 0.001**
Branch leaves	0.085	**< 0.001**	0.091
All leaves	0.828	**< 0.001**	**0.024**

[a] Open or dense stand. [b] Leaf number, area, dry mass or nitrogen. [c] *P*-values are presented; **bold** for $P < 0.05$.

Table S4. Specific respiration rates (mg CO_2 g^{-1} h^{-1}; 25°C) of leaf, stem, root, and the reproductive part in *Xanthium canadense* plants grown in an open (6.25 plants m^{-2}) or in a dense stand (59.2 plants m^{-2})

Date[a]	Leaf	Stem	Root	Reproductive
Open stand				
19 (July)	2.37 (0.60)[b]	2.93 (0.33)	3.22 (1.08)	-
48 (Aug)	1.73 (0.26)	1.34 (0.24)	1.83 (0.58)	-
74 (Sept)	1.10 (0.16)	0.57 (0.10)	0.70 (0.10)	3.78 (0.67)
105 (Oct)	1.39 (0.19)	0.41 (0.05)	0.47 (0.07)	0.70 (0.06)
137 (Nov)	-	0.34 (0.22)	0.65 (0.13)	0.02 (0.01)
Dense stand				
19 (July)	2.50 (0.77)	2.95 (0.55)	3.13 (1.39)	-
48 (Aug)	1.37 (0.25)	1.15 (0.27)	1.26 (0.16)	-
74 (Sept)	1.34 (0.51)	0.40 (0.05)	0.55 (0.07)	2.54 (0.26)
105 (Oct)	2.98 (0.63)	0.49 (0.11)	0.64 (0.14)	0.77 (0.23)
137 (Nov)	1.48 (-)	0.30 (0.21)	0.82 (0.31)	0.11 (0.18)

[a] Date of the first measurement in the month (parentheses), in days after transplanting (23 June 2011). [b] Values are means and s.d. in parentheses.
